# Inconsistencies of *Gaussian's* “*NBO 3.1*” Module With Authentic Natural Population Analysis

**DOI:** 10.1002/jcc.70374

**Published:** 2026-04-12

**Authors:** Frank Weinhold

**Affiliations:** ^1^ Theoretical Chemistry Institute and Department of Chemistry University of Wisconsin‐Madison Madison Wisconsin USA

**Keywords:** atomic charge, discrepant “NBO analysis”, *Gaussian 16* “NBO 3.1” module, heavy‐atom metallicity, natural population analysis (NPA)

## Abstract

We provide illustrative examples of the discrepancies between “NBO analysis results” as obtained from *Gaussian‐16*'s proprietary *NBO 3.1* module versus the authentic *Natural Bond Orbital* program as first developed in the 1980s and maintained in the current *NBO7* program version.

## Graphical abstract

Erroneous NPA atomic charge values from *Gaussian 16's* “NBO 3.1” module (red) are replaced by proper {*Q*_Hg_} NPA values (yellow) in rhombohedral Hg_13_ unit cell model of superconducting mercury.

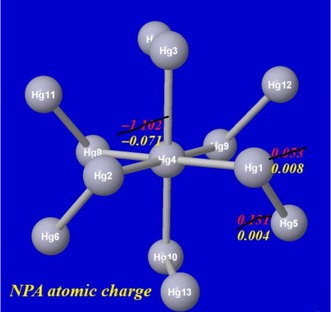

## 1 Introduction

A recent paper [1] claiming what was described as “direct evidence for a carbon‐carbon one‐electron bond” was critically re‐examined in subsequent work that disputes this claim [2]. Both papers offer theoretical analysis based on what is described as natural bond orbital (NBO) methodology [3], but the resulting conclusions differ in both quantitative and qualitative respects [4]. These inconsistencies largely trace to Reference [1] usage of “*NBO 3.1*” as incorporated in the *Gaussian 16* (*G16*) program system [5], rather than the authentic NBO algorithms as first developed by the author nearly a half‐century ago and subsequently distributed in successive versions up to current *NBO7* [6].

Unfortunately, such conflicting representations of “NBO analysis” are rather common. Although early tests of *NBO 3.1* numerics for systems of a few light atoms and near‐minimal basis orbitals gave approximate agreement with then‐current *NBO3* counterparts, such agreement is by no means assured in typical computational applications of the current literature. Numerous literature studies can now be recognized that present what are described as “NBO results” based on the invalid numerics of the *Gaussian 16* implementation, with corresponding citation of the present author as responsible for the results and conclusions.

This work briefly presents illustrative examples of the major differences that can arise between purported NBO analysis results from the *NBO 3.1* module of the current *Gaussian 16* distribution versus those from the original *NBO 3* and ensuing program versions *NBO 4‐7*. The goal is to alert current *G16* users to the inherent ambiguities and inconsistencies of expecting *NBO 3.1* numerical results to be equivalent to those of authentic NBO analysis.

## 2 Early History of NBO Development

The first conception and numerical applications [7, 8] of what came to be known as NBO methodology were carried out in the framework of John Pople's original *Gaussian* host program of the mid‐1970s. The Fortran code for Pople's program appeared as the Appendix of his *Approximate MO Theory* book [9] based on the semi‐empirical assumptions of then‐current LCAO‐MO methods. As numerical methods advanced for accurate evaluation of 1*e*/2*e* integrals with Gaussian‐type orbitals, the foundation was laid for fully ab initio versions of the *Gaussian* program system as we know it today [10].

Successive “open access” *Gaussian XX* versions (*XX* = 70, 76, 80, 82, 86, 88, 90, 92, 94, 98) consistently included interfacing to the *NBO* code as developed in extended collaborations between Pople and Weinhold research groups, originally distributed through the Quantum Chemistry Program Exchange (QCPE) at Indiana University. However, this code was subsequently modified and relabeled “*NBO 3.1*” for inclusion in initial proprietary releases of *Gaussian 03* [11] and ensuing *Gaussian 09* and *Gaussian 16* versions as commercial *Gaussian Inc* products, without the consent or approval of the NBO developers [12].

We consider the *NBO7* program to be the authentic “voice” of NBO concepts and their ongoing development and usage. Details of the source‐code modifications of *NBO 3.1* made by post‐Pople *Gaussian* developers are now unavailable to outsiders. Few options are available to communicate the overall scope of differences to the broader community of nontheoretical specialists who may employ *NBO 3.1* numerical results without adequate awareness of these modifications or proper identification of the employed software.

Of course, current *NBO7* includes numerous features beyond those of earlier *NBO 3.0/3.1* program versions. Chief among these are the natural resonance theory (NRT) [13, 14] options that have largely superseded NBO theory as the fundamental conceptual framework for describing electronic phenomena in localized chemical bonding terms, including the sub‐integer fractional bond orders of the supramolecular domain [15]. *NBO 3.1* provides no counterpart for these modern NRT developments.

*NBO 3.1* is most directly compared with modern *NBO7* in terms of the natural atomic orbitals (NAOs) and occupancies, particularly the natural atomic charges {*Q*_A_} of natural population analysis (NPA) [16]. Determination of NPA atomic charges involves no dependence on subsequent steps of NBO formation or other constructions of NBO/NRT analysis. However, any significant *NBO 3.1*/*NBO7* difference in *Q*_A_ values leads to a cascade of differing results throughout program output. We therefore focus exclusively on {*Q*_A_^(3.1)^} versus {*Q*_A_^(7)^} NPA differences in the illustrative examples to follow.

## 3 Illustrative Examples of *NBO 3.1*/ *NBO7* Differences

**FIGURE 1:**
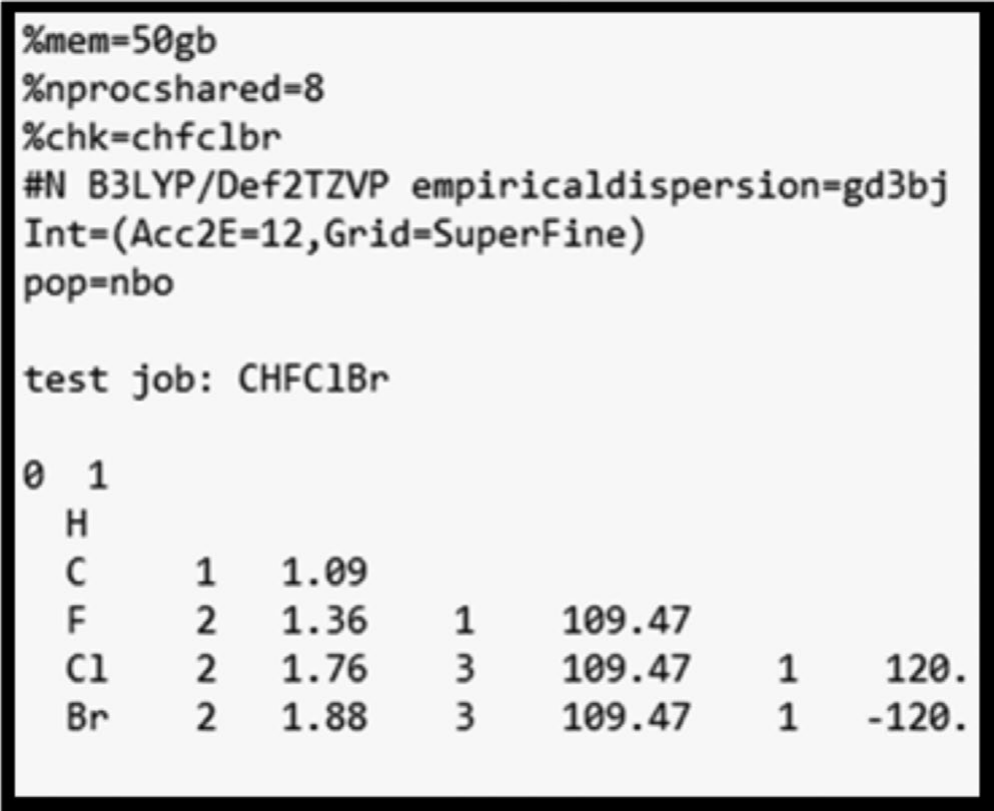
Sample *G16* input file to obtain *NBO 3.1*‐level NPA output for the test job. Changing “pop = nbo” to “pop = nbo7” (for a tandem *G16/NBO7* installation) yields authentic *NBO7* NPA output.

For simplicity and consistency, we employ the same density functional‐theoretic (DFT) methodology and basis set for each system considered below. Figure 1 displays the *G16* route‐card specification and file‐format that is used throughout, with the common choice of Grimme‐corrected B3LYP functional, Def2TZVP basis set, and highest available numerical precision for all integral evaluations. The examples below are chosen (rather arbitrarily) to sample a variety of atomic weights, electronegativities, periodic groups, ionic character, and structural departures from equilibrium. The interested reader is invited to repeat these comparisons of {*Q*_A_^(3.1)^} and {*Q*_A_^(7)^} atomic charges for any alternative choice of DFT/wavefunction method, basis set, or relativistic correction in common usage, or for other choices of the chemical system of interest.

### 3.1 Borodin‐Hunsdiecker Reaction Complex

The first example is drawn from the transition‐state region of the Borodin‐Hunsdiecker reaction [17], involving decarboxylation of silver acetate by dibromine to yield AgBr, CH_3_Br, and free CO_2_:

(1) $${\mathrm{CH}}_{3}\text{COOAg}+{\mathrm{Br}}_{2}\to \text{AgBr}+{\mathrm{CO}}_{2}+{\mathrm{CH}}_{3}\mathrm{Br}$$

**FIGURE 2:**
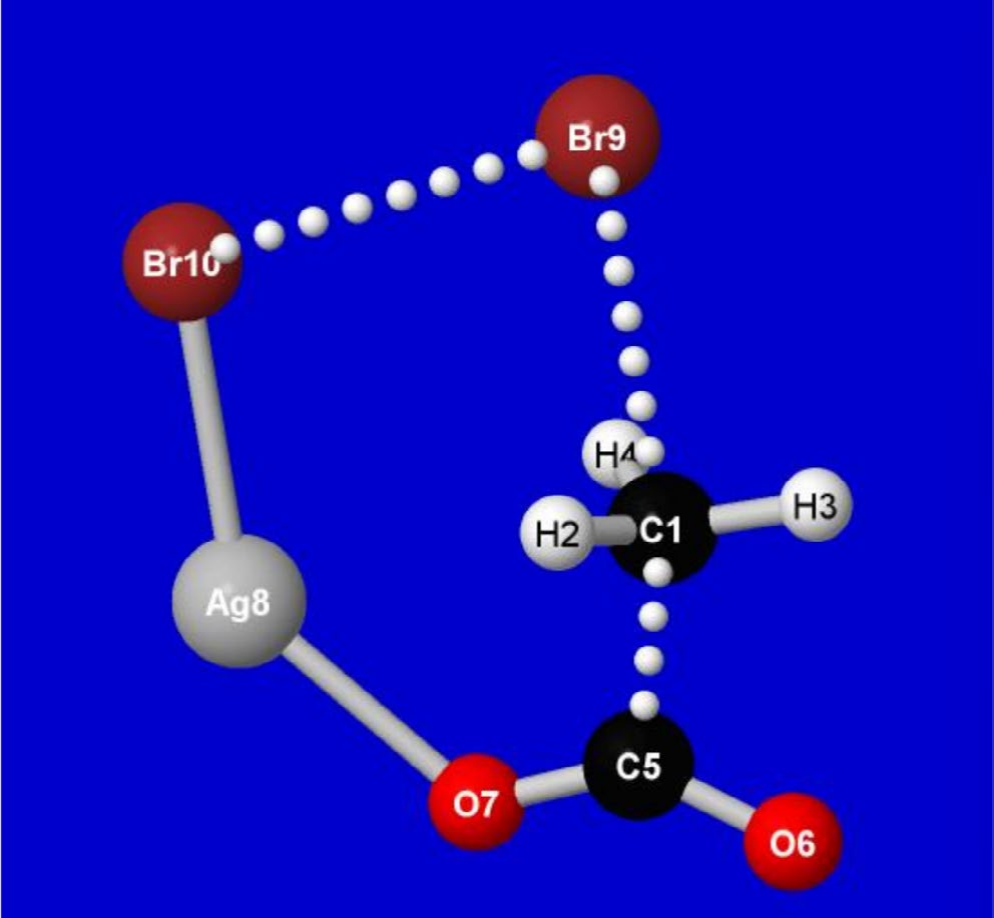
Snapshot of the methyl‐transfer transition‐state region of model Borodin‐Hunsdiecker decarboxylation reaction (1).

As shown in Figure 2, the snapshot geometry exhibits the coordinated Br_2_ bond‐breaking and methyl transfer of this far‐from‐equilibrium region, where changes of charge character may be occurring at multiple sites of the transition‐state complex. This complex includes the usual C/H/O atoms of common bioorganic species, as well as less‐common heavy halogen Br and transition‐metal Ag atoms, all in coordinated transfers that make it less obvious how to “intuit” their likely atomic charges at this point of the reaction pathway.

Table 1 displays the comparison of calculated *NBO 3.1* and *NBO7* atomic charges for each atom of the Borodin‐Hunsdiecker complex, with the qualitative magnitude and sign of the percentage error (with respect to the *NBO7* entry) shown in the final column.

**TABLE 1:** Comparison {*Q*_A_^(3.1)^} vs. {*Q*_A_^(7)^} NPA differences (and percentage errors of the former) for atoms of the Borodin‐Hunsdiecker reaction pathway (Figure 2).

Atom	NBO 3.1	NBO7	%‐error
C 1	−0.64049	−0.64750	+1%
H 2	+0.25121	+0.25354	−1%
H 3	+0.24486	+0.24544	< 1%
H 4	+0.25121	+0.25354	−1%
C 5	+0.81244	+0.81114	< 1%
O 6	−0.45166	−0.45384	< 1%
O 7	−0.64014	−0.66321	+3%
Ag 8	+0.68012	+0.76633	−11%
Br 9	−0.13876	−0.13846	< 1%
Br 10	−0.36880	−0.42700	+14%

For the common main‐group C/H/O atoms, the agreement is generally seen to be quite good (ca. 1%), with small deviations that may shift in positive or negative direction from the *NBO7* value. For the Br(9) atom the agreement is similarly close, but both Br(10) and Ag(7) exhibit significantly larger (> 10%) errors, where even the first fractional digit is likely to disagree between calculated *NBO 3.1* and *NBO7* values. This example serves notice of the more severe discrepancies between {*Q*_A_^(3.1)^} and {*Q*_A_^(7)^} values that may appear when applications beyond small near‐equilibrium organic species are considered.

### 3.2 YBCO Superconductor Half‐Cell

**FIGURE 3:**
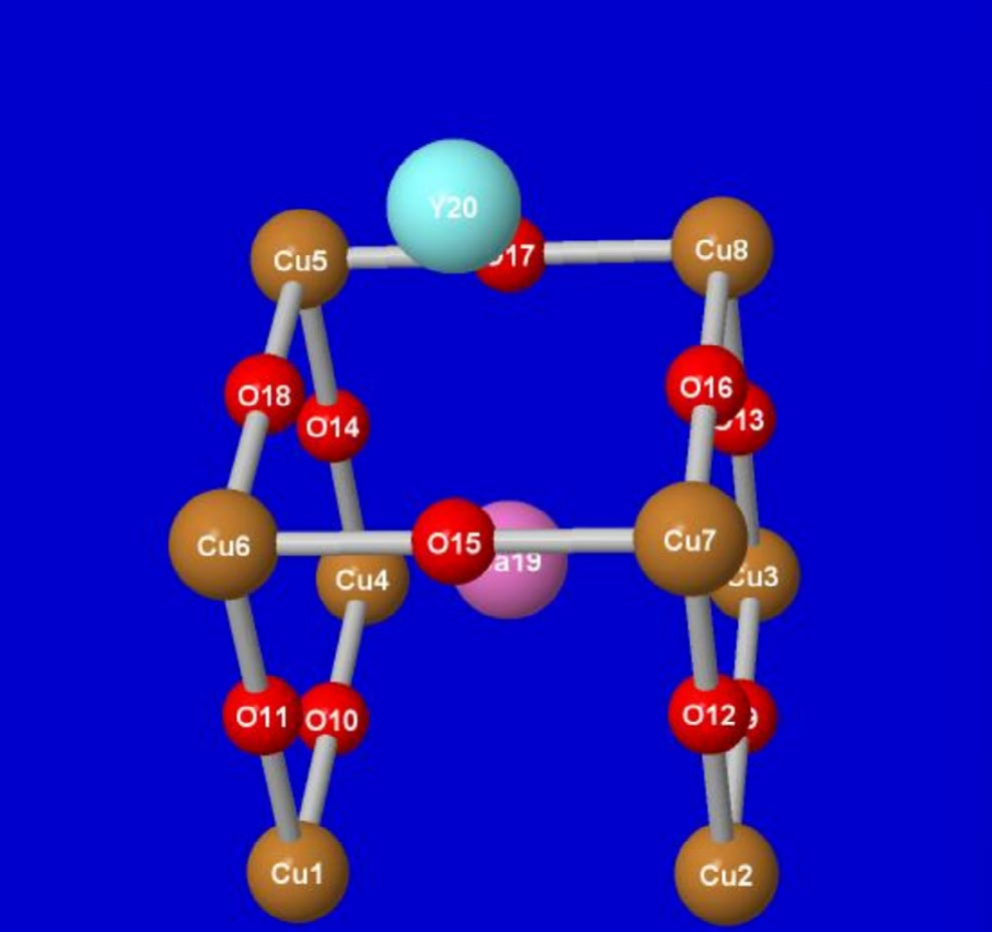
(Slightly) idealized model of lower‐half of the experimental unit‐cell geometry of YBCO superconductor [11].

A second example is chosen as an idealized theoretical model for (one‐half of) the unit cell of the high‐temperature YBCO cuprate superconductor [18]. Figure 3 displays the atomic numberings and qualitative structural features of the unit‐cell cluster, with model lattice parameters taken from experimental values. The 20‐atom model features uncommon barium [Ba(19)] and yttrium [Y(20)] atoms as well as eight Cu and ten O atoms of the cuprate lattice. As shown in the figure, four of the eight Cu atoms and seven of the ten O atoms occupy symmetry‐unique sites where distinct charge variations from idealized fixed “cuprate” values might be found.

Table 2 displays the comparisons of {*Q*_A_^(3.1)^} versus {*Q*_A_^(7)^} atomic charges for each of the 13 symmetry‐unique atoms of the YBCO unit cell. Despite the dominant number (seven) of “common” O atoms, the %‐error deviations show significant percentage errors (ca. 10%–15%) from authentic *NBO7* values at virtually all sites of the YBCO complex. Differences of this magnitude are likely to be off‐target even in the first fractional digit of each *Q*_A_^(3.1)^ estimate.

**TABLE 2:** Similar to Table 1, for atoms of the model YBCO half‐cell of Figure 3.

Atom	NBO 3.1	NBO7	%‐error
Cu 1	+0.89603	+0.98598	−9%
Cu 2	+0.90618	+0.99333	−9%
Cu 5	+0.96861	+1.15296	−16%
Cu 7	+0.99032	+1.17251	−16%
O 9	−1.18701	−1.27111	+7%
O 10	−1.20590	−1.29367	+7%
O 11	−0.87402	−0.97553	+10%
O 12	−0.91092	−1.01067	+10%
O 15	−1.21018	−1.35334	+11%
O 16	−1.12533	−1.25637	+10%
O 18	−1.14988	−1.28453	+10%
Ba 19	+1.80318	+1.72318	+5%
Y 20	+1.33292	+1.45203	−8%

The results of Table 2 show that even common O atoms of the organic regime may incur much larger discrepancies between *NBO 3.1* estimates and actual NPA {*Q*_O_^(7)^} values. In the seven examples of Table 2, the {*Q*_O_^(3.1)^} values are found to be consistently more cationic (+) than {*Q*_O_^(7)^} values. However, the variations in magnitude (7%–11%) indicate that no simple proportionality factor could bring {*Q*_O_^(3.1)^} values back into close correlation or agreement with {*Q*_O_^(7)^} values, as appeared in Table 1. The variations in {*Q*_Cu_^(3.1)^} error‐magnitudes for the four Cu atoms (9%–16%) indicate their similarly irregular behavior relative to authentic {*Q*_Cu_^(7)^} values.

### 3.3 Unit Cell of the Superconducting Phase of Metallic Mercury

**FIGURE 4:**
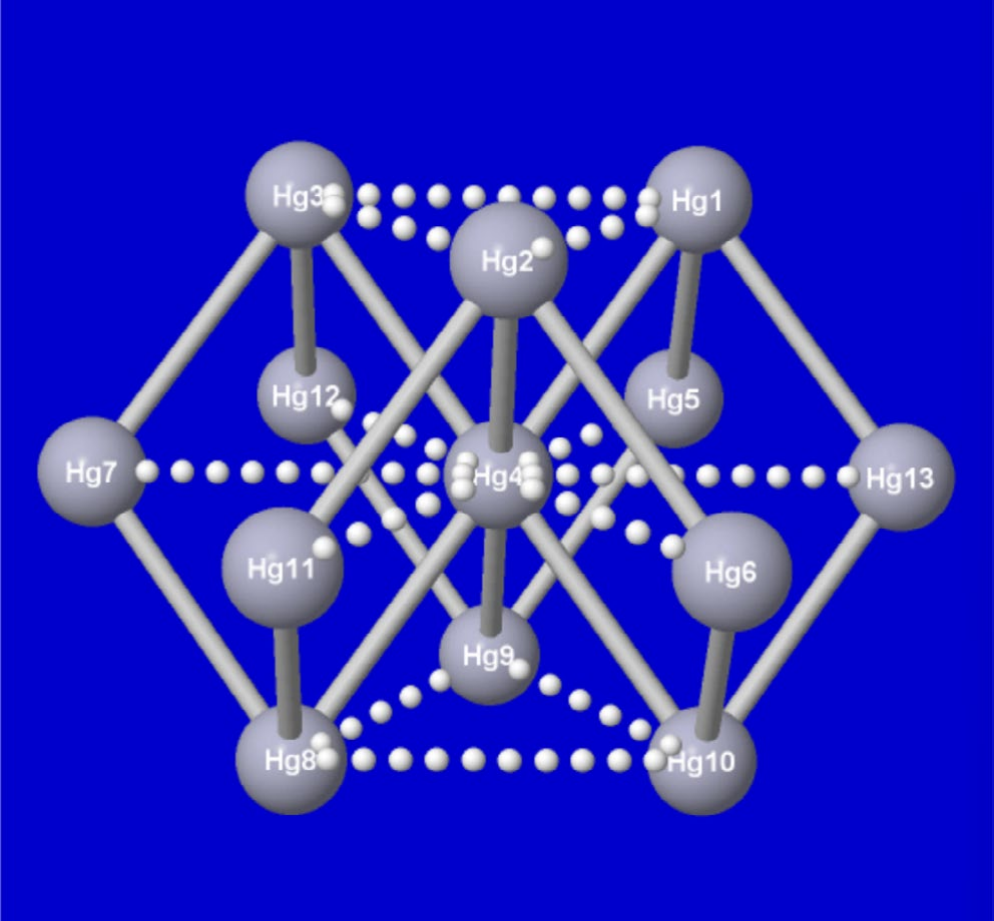
*D*_3d_‐symmetric Hg_13_ metallic mercury cluster, displaying the numbering for symmetry‐unique central [Hg(4)], mid‐plane [Hg(5)], and top [Hg(1)] sites of the Hg_13_ cluster.

Finally, we consider the calculation of atomic charges for a pure mercury Hg_13_ cluster (Figure 4), the 13‐atom unit cell of the low‐temperature superconducting phase of metallic mercury [19]. Elemental mercury is the material in which the phenomenon of superconductivity was first discovered, signaling a type of electronic mobility that seems contrary to its apparent nobility at the end of the 5*d* block.

As shown in Figure 4, the Hg_13_ cluster is of unusual rhombohedral (*D*_3d_) symmetry, with only three of the thirteen Hg atoms at symmetry‐unique sites. Superficially, this cluster has no differences in atomic electronegativity, size, or periodic assignment of the compared sites, suggesting that this species offers the “easiest” test of consistency between calculated *NBO 3.1* and *NBO7* atomic charges.

However, Table 3 displays calculated {*Q*_A_^(3.1)^}, {*Q*_A_^(7)^} values for Hg_13_ that demonstrate this expectation of consistency to be erroneous. The three distinct sites are seen to exhibit *order‐of‐magnitude* differences from one another. More importantly, the *Q*_Hg_ values as calculated by *NBO 3.1* differ qualitatively (by factors of 5–30!) from those calculated by *NBO7*. Apparently, the former cannot be trusted to give even *order‐of‐magnitude* agreement with authentic *NBO7* values in this case.

**TABLE 3:** Similar to Table 1, for the symmetry‐unique mercury atoms of the Hg_13_ cluster (Figure 4).

Atom	NBO 3.1	NBO7	%‐error
Hg 1	+0.05320	+0.00814	+554%
Hg 4	−1.10222	−0.07131	−1446%
Hg 5	+0.13050	+0.00375	+3380%

More generally, it appears that the modifications made in *NBO 3.1* lead to increasingly catastrophic failures to emulate the authentic *NBO7* algorithms as one moves to heavier atoms outside the focus of mid‐1980s studies of simple organic species. The few examples quoted here can only begin to suggest the extent of these problems throughout the domain of heavier metallic elements.

## 4 Conclusion

Differing numerical results from the “*NBO 3.1*” module of current *Gaussian 16* (*G16*) program [5] versus authentic (NPA) values from the current *NBO7* program [6] are significant and concerning. *G16* users should be aware of these discrepancies and provide clear citation for what is quoted as “NPA/NBO results” from this platform, if not properly configured with tandem *G16/NBO7* co‐programs.

## Conflicts of Interest

The author declares no conflicts of interest.

## Data Availability

Data sharing not applicable to this article as no datasets were generated or analyzed during the current study.
